# Letter from K

**Published:** 1854-01

**Authors:** 


*Prof. Taylor.*—DEAR SIR; We, of the professional brotherhood, who dwell deep within the wilds of the far South and West, would have a lonely time in our travels from one village to another, in our circuit, if we did not occasionally meet with "*cha-rac-ters*" serving for amusement and instruction.

Crossing the Choctawhatchic river, in one of my late "*towers*," I was irresistably drawn into conversation with the ferryman; and very soon he had "pumped" from me my name, residence, and that I was a "*tooth doctor*." "Tooth doctor, aha! I've got mighty good teeth, never had toothache but once in my life, couldn't get it drawd. I'll tell you what will cure toothache *certing*, cured mine; just killed it dead as a hammer, brought it right out." "You see I had the toothache *powerful*, I didn't know what to do." "Somebody told me to put rattlesnake's grease in it, so I took a piece of cotton, and sobbed it in rattlesnake's grease, and put it in the hollow, and it cured it. Tell you sir its a mighty sure remedy, but it is *nasseous* for true, and a man must have the toothach powerful bad, must be in a great rack of misery, and have a strong stomach, before he can go it."

Place this "grease" among your odontalgic remedies, and let the juniors of the profession, find in the following the reason their fillings sometimes fall out, and cease to be desponding and unhappy.

There is hidden away in these wild woods, a *dentist*, who, by dint of low price and hard persuasion, got a few teeth submitted to his manipulations. With great dispatch he patched the faulty places, and promised his *ears* if the fillings came out. But alas, for human calculations; only a short time passed, and the fillings were missing and the ears demanded. "Stop, *stop*! haven't your tooth been sore? Haven't you had a cold?" Yes, b'lieve I have. "Well then I'm not responsible." You see, should you take cold, and the teeth are sore—they *swell*, and the *hole* gets larger than the plug; there's nothing to hold it in, and its bound to come out."

His patients were edified and satisfied. K.

P. S. The "specimen" who furnisher the above fact, says he has read Harris' "*surgal dentry*."

